# Understanding core competencies of older adult care workers from the perspectives of the employer: automated content analysis of job advertisements

**DOI:** 10.3389/fpubh.2026.1847475

**Published:** 2026-08-18

**Authors:** Meiling Chen, Weijue Wang, Meizhen Wu, Chunyan Jing

**Affiliations:** 1 Institute of Zhejiang Chinese Medical Culture, Zhejiang Chinese Medical University, Hangzhou, China; 2 School of Humanities and Management, Zhejiang Chinese Medical University, Hangzhou, China

**Keywords:** China, competence, content analysis, older adult care, job advertisements

## Abstract

**Objective:**

This study aimed to examine employer-defined core competencies for older adult care workers in China.

**Methods:**

From June to July 2025, we scraped 3,068 job advertisements for older adult care positions across 15 Chinese cities from three popular Chinese online recruitment sites. Based on the Iceberg Competency Model, we designed an analytic framework, which was then analyzed via automated content analysis (DivoMiner platform).

**Results:**

The employer-stated competency requirements for older adult care workers were ordered hierarchically. At the skill level, the most significant competencies were activities of daily living support (personal hygiene: 70.24%; household assistance: 62.71%), supplemented by growing expectations for health-related care (health monitoring: 41.85%; rehabilitation care: 21.42%) and psychological support (emotional companionship: 26.21%). Regarding comprehensive abilities, soft skills, communication (41.23%), patience (31.91%), and compassion (29.11%) were highly valued. The positive predictors of high salaries for older adult care workers were urban economic level [*B* = 0.770, OR = 2.160, 95% confidence interval (CI): 1.890–2.468, *P* < 0.001], compassion (*B* = 0.409, OR = 1.505, 95% CI: 1.230–1.842, *P* < 0.001), communication skills (*B* = 0.389, OR = 1.476, 95% CI: 1.215–1.791, *P* < 0.001), and mealtime assistance skills (*B* = 0.316, OR = 1.372, 95% CI: 1.099–1.712, *P* = 0.005).

**Conclusion:**

The employer-expressed competency demands for older adult care workers have gradually expanded from daily living support to a multi-functional role covering daily living, health care, and psychological care. Soft skills and humanistic care literacy are key to evaluating the comprehensive competence of older adult care workers and vital for securing high salaries. Our findings provide empirical evidence for older adult care education curriculum design, talent evaluation systems, and policymaking.

## 1 Introduction

An aging population is a global public health issue. According to the World Population Prospects of the United Nations 2024 (1), people aged ≥65 years currently account for 10% of the global population (approximately 830 million) and are projected to double to nearly 20% by 2050. China is one of the rapidly aging countries worldwide. Recent data show that the population of individuals aged ≥65 years is over 220 million, accounting for 15.6% of the population, which is expected to increase by 26% by 2050 (2). The continuous increase in the proportion of the older population, especially among those aged ≥80 years, significantly increases the demand for long-term care (LTC) services (3–5).

Older adult care workers are the core human capital who directly provide daily care, professional nursing services, and emotional support to older adults, and their competence directly affects the quality of life, safety, and dignity of older adults. Studies have shown that a standardized competency-based framework can mitigate role ambiguity and optimize resource allocation and service monitoring, thereby improving the quality of older adult care globally (6). It can also foster a stronger professional identity and enhance job retention among older adult care workers (7). Cleland et al. presented a systematic literature review and recognized nine dimensions for older adult care quality, which are respect and dignity, identity support, older adult care workers' skills, relationships with older adult care workers, informed choices, social relationships and the community, supporting the health, wellbeing, safety, comfort, ability to make complaints and provide feedback to the aged care organization (8). The Australian Geriatric Nursing Competencies, established and validated by Traynor et al., encompass modern dimensions such as ethics, law, and technology, culminating in 11 core competencies (9). Collectively, these findings indicate a research consensus on improving care quality through a standardized competency framework. However, existing research still has deficiencies. Most research focuses on optimizing the training system on the supply side or on policy analysis, lacking a systematic examination of the actual needs of employers (10). Methodologically, it primarily relies on small-sample questionnaire surveys or case interviews, making it difficult to capture large-scale market dynamics (11).

Job advertisements are considered a practical information source for researchers, educators, policymakers, and higher education institutions to explore the job market dynamics (12). They outline the role expectations of the candidate and the responsibilities, skills, knowledge, behaviors, traits, or characteristics that are considered necessary to select the right candidate (13). Online job advertisements offer significant advantages over traditional research methods as an innovative data source for researching occupational competence. Online job information is highly time-sensitive, enabling timely reflection of the dynamic changes in labor market demand for skills and qualifications and effectively avoiding the inherent time lags of questionnaire and interview data (14). Online job advertisements also substantially reduce research costs and minimize subjective biases (15). Using web crawlers and automated text analysis techniques, researchers can efficiently and cost-effectively collect and process large-scale recruitment data. Since recruitment content directly reflects the real hiring needs of employers rather than the subjective self-reports and recollections of respondents, it can more accurately portray the actual requirements of the labor market for occupational competence.

This study examines the core competencies required of older adult care workers from the perspective of the employers by analyzing 3,068 job advertisements collected from 15 major Chinese cities between June and July 2025. The study aims to (1) identify the core job skills that employers consider when recruiting older adult care workers and (2) explore the key factors influencing high salary attainment for older adult care workers as reflected in the sampled job advertisements. The research results provide empirical evidence for older adult care education course design, talent assessment system development, and policymaking.

## 2 Methods

This study primarily employs automated content analysis. Automated content analysis is a technique that uses computer technology and natural language processing to analyze large volumes of text data. This method can significantly improve efficiency, reduce errors in manual coding, and reduce labor intensity (16, 17). This study employed the text data mining analysis platform, DivoMiner, as an automated content analysis tool (18). DivoMiner is a cloud-based text big-data mining and analysis platform. This platform integrates traditional content analysis with modern artificial intelligence technologies, enabling the entire process online, including text content classification, semantic judgment, reliability testing, coding (manual and NLP), and generating visual charts (19). The analysis was divided into four steps: sample selection and data acquisition, analysis framework and codebook development, machine coding, and statistical analysis.

### 2.1 Sample selection and data acquisition

Stratified sampling was used to ensure a representative sample. Based on the per capita gross domestic product data from the National Bureau of Statistics in 2024, all prefecture-level and above cities in the country were classified into three tiers by economic development level: high, medium, and low. Five cities were randomly selected from each tier, and 15 sample cities were ultimately determined, including Beijing, Guangzhou, Hangzhou, Nanjing, Shanghai, Chengdu, Hefei, Xi'an, Zhengzhou, Changsha, Harbin, Shijiazhuang, Guiyang, Taiyuan, and Changchun.

**Figure 1:**
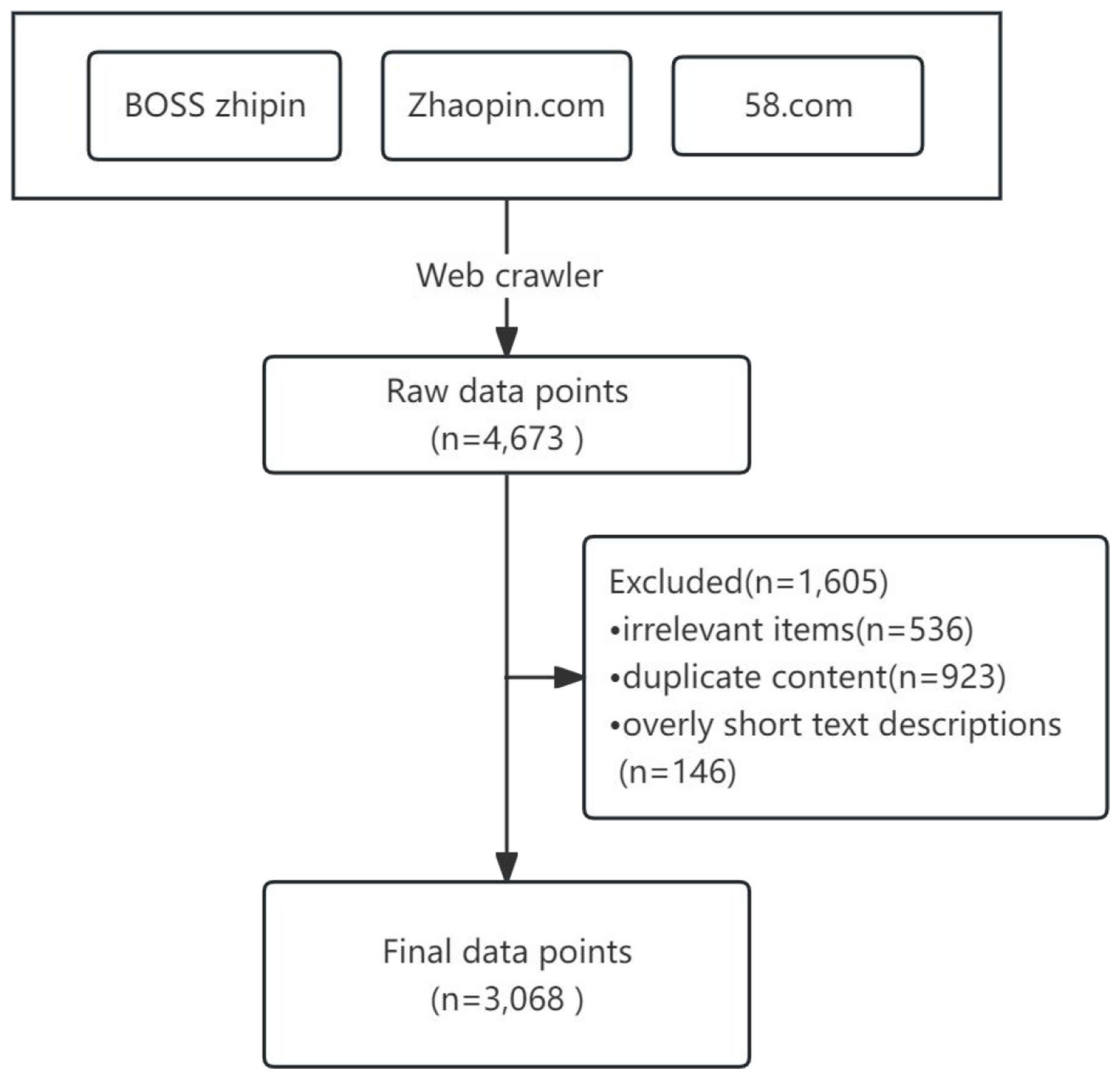
Data inclusion flow chart.

The data inclusion process is shown in Figure 1. The data for this study were sourced from the three major domestic online recruitment platforms: BOSS Zhipin, 58.com, and Zhaopin.com. These three platforms were selected because they have broad user coverage and high enterprise activity, which better reflect the demand for older adult care workers in the Chinese labor market, thereby ensuring the representativeness and external validity of the research sample.

Data were collected using Octoparse for web scraping. Two Chinese keywords, namely “养老护理员” (Older adult Care Worker) and “老年照护” (Aged Care), were adopted for information retrieval. We scraped relevant job recruitment postings released in the aforementioned 15 sample cities, with the data collection period spanning from June 25 to July 16, 2025. Initially, 4,673 raw data points were collected. The inclusion criteria required job postings to explicitly recruit for older adult care workers/aged care positions, have complete job description content, and be located in the 15 predefined sample cities. Exclusion criteria removed irrelevant job postings not related to older adult care (e.g., general nurse positions), duplicate postings from the same employer with identical job content, and postings with job description text shorter than 20 Chinese characters that failed to provide sufficient competency-related information. Deduplication rules retained only the first scraped record for postings with identical job title, recruiting organization name, salary level, and job description content. The collected information mainly included job title, work experience, posting city, educational requirements, job description, recruiting organization size, and salary level. Excel 2016 (Microsoft Corp.) was used to clean and preprocess data: irrelevant items, duplicate content, and overly short text descriptions were removed, and structured data, such as work location, work experience, educational requirements, and salary, were uniformly organized and categorized. Among them, the salary ranges were standardized to average monthly salaries. Finally, 3,068 valid data points were obtained.

### 2.2 Analytical framework and codebook development

The first step in content analysis is selecting a unit of analysis. Job descriptions in recruitment advertisements were chosen as the unit of analysis. The analytical framework of this study is based on the Iceberg Model of Competencies (20). The Iceberg Model categorizes individual competencies into two types. The first comprises explicit, above-water characteristics, including professional knowledge and practical skills. The second encompasses implicit, below-water characteristics, such as general abilities, personality traits, motivation and values. This model emphasizes the decisive role of deep psychological traits in shaping individual behavioral performance (21). With its clear, hierarchical structure that integrates both surface and deep competency elements, the Iceberg Model offers unique advantages for nursing competence research. In recent years, scholars have developed multiple competency evaluation systems for specialized nurses based on this model. Relevant applications include frameworks for liver transplant specialist nurses (22), pain resource nurses (23), and assessment scales for dementia care nurses (24). These studies encompass multidimensional indicators ranging from professional knowledge to intrinsic motivation. Their scientific validity and practical utility have been systematically validated. This study integrates it with the “National Vocational Skill Standards for Older Adult Care Workers (2019 Edition)” issued by the Ministry of Human Resources and Social Security of the People's Republic of China to ensure that the classification system is both theoretically forward-looking and policy-compatible (25). This enables the theoretical framework to align with the actual norms and specific requirements of the Chinese older adult care industry.

**Figure 2:**
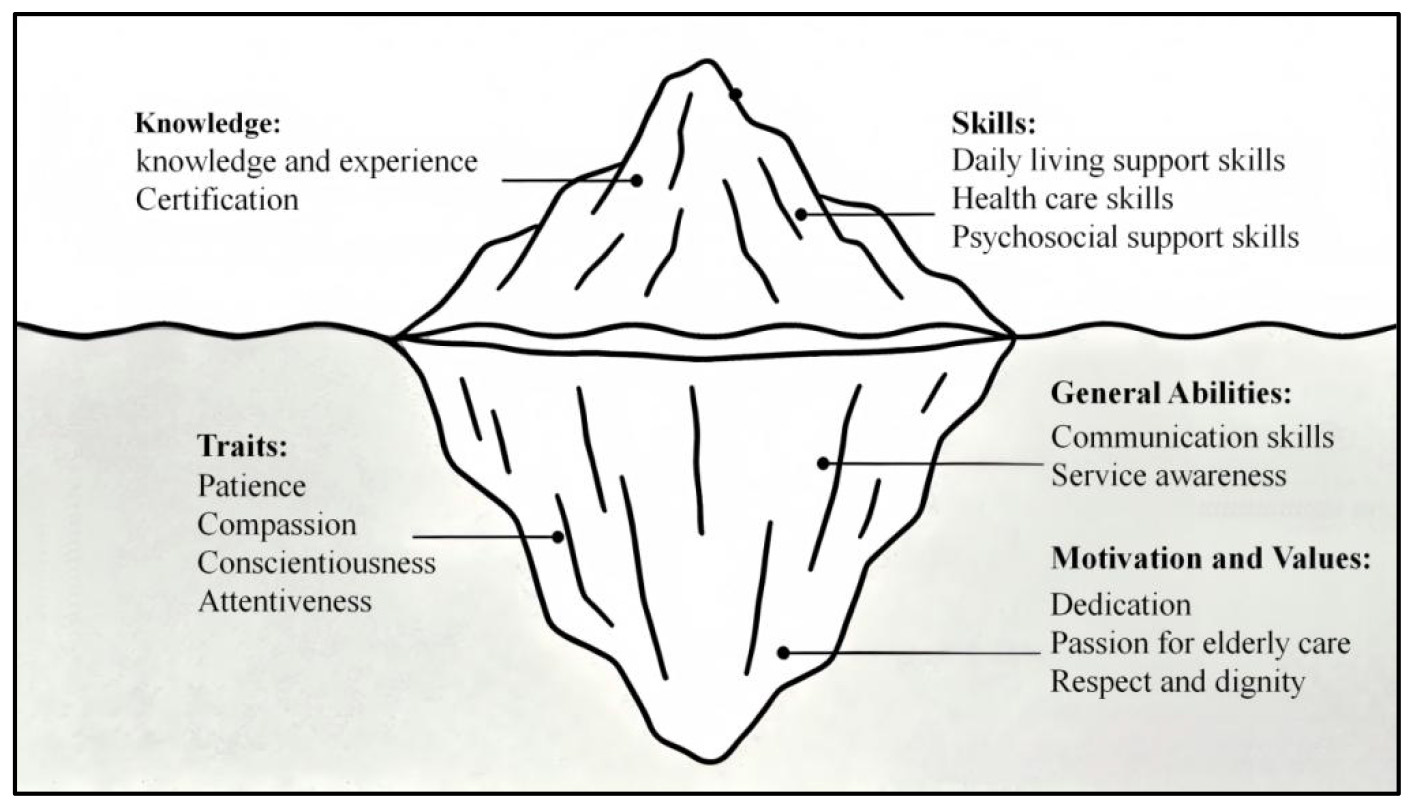
Iceberg model of competency for older adult care workers.

Consequently, five core dimensions were established along with their operational definitions (Figure 2).

- Knowledge: refers to the professional care knowledge necessary for engaging in older adult care work, the required working years, and the nationally recognized qualification certificates.
- Skills: encompass the practical operational abilities required to complete specific care tasks, including daily living support, healthcare, and psychosocial support skills.
- General abilities: represents advanced generic professional competencies, including communication skills and service awareness.
- Traits: focuses on the enduring psychological attributes of the employees, including patience, compassion, conscientiousness, and attentiveness.
- Motivation and values: represent the fundamental motivators of behavior, including dedication, passion for older adult care, and the value of upholding the respect and dignity of older adults.

### 2.3 Machine coding

We used DivoMiner to implement the machine coding. During the machine encoding stage, the previously constructed five-dimensional competency framework was first transformed into a structured keyword dictionary. Specifically, the research team conducted a pre-analysis of some samples, extracting, summarizing, and identifying the core keywords and their synonymous expressions in each dimension. For instance, in the communication ability dimension, various expressions such as “good at communicating,” “strong communication ability,” and “companionship chatting” were included to ensure that the dictionary had good coverage and semantic inclusiveness. Subsequently, all 3,068 valid texts were scanned automatically, and multivalued coding was applied using the preset keywords.

A total of 153 texts (5% of the total sample size) were randomly selected. Two trained manual coders independently conducted the coding to assess the coding. The agreement between machine coding and manual coding was assessed using Cohen's Kappa coefficient. The results revealed a kappa value exceeding 0.90, indicating an almost perfect level of agreement and affirming the dependability of the machine coding method.

### 2.4 Statistical analysis

DivoMiner and Statistical Package for the Social Sciences (SPSS) (IBM SPSS 25.0, SPSS Inc.) software were used to analyze the coding data, including frequency statistics, percentage calculations, and analysis of variance (ANOVA), to reveal the explicit demand intensity and distribution.

Binary logistic stepwise forward regression was used to identify the key factors influencing the attainment of high salaries among older adult care workers. The study variables were defined as follows: the dependent variable was “high salary,” which was binary; samples with monthly salaries in the 75th to 100th percentile for older adult care workers were coded as 1 (high-salary group,monthly salary>8,000), and those in the remaining percentiles as 0 (non-high-salary group). The independent variable system comprised 21 core skill indicators for older adult care positions. Urban economic level, educational requirements, and work experience were also included as confounding variables to control for potential confounding effects of external environmental characteristics and job-entry thresholds on salary levels.

## 3 Results

### 3.1 Source of job advertisements and employer information

As presented in Table 1, job postings for older adult care positions were predominantly sourced from three major platforms: BOSS Zhipin (62.78%), 58.com (19.04%), and Zhaopin (18.19%). The distribution of position numbers is unbalanced between regions. The number of positions in high-economic-level regions is the highest (51.79%), followed by medium-economic-level regions (28.78%), and then low-economic-level regions (19.43%). In the older adult care service sector, organizations with 21–99 employees made up the largest portion at 33.80%, with those having 1–20 employees next at 22.39%. This distribution showed that the older adult care service industry is predominantly composed of small and medium-sized enterprises.

**Table 1:** Sources of job advertisements and employer information.

Variable	Category	Frequency	Percentage (%)
Source	BOSS Zhipin	1,926	62.78
	58.com	584	19.04
	Zhaopin.com	558	18.19
Urban economic level	High	1,589	51.79
	Medium	883	28.78
	Low	596	19.43
Organizational size	21–99 employees	1,037	33.80
	1–20 employees	687	22.39
	Unreported	601	19.59
	100–499 employees	460	14.99
	500 or more employees	283	9.22
Total		3,068	100.00

### 3.2 Basic job thresholds and salary levels

As presented in Table 2, the barriers to entering older adult care positions are relatively minimal, with limited prerequisites regarding work experience and education. Positions labeled “No Experience Required” made up 76.92% of the total, far exceeding those requiring “1–3 years of experience” at 14.41%. Regarding educational qualifications, positions with “no minimum requirement” have the highest proportion, reaching 79.92%. These positions mainly involve basic care. Positions necessitating a “junior high school education or below” constituted 10.50%, and typically required proficiency in essential care skills, emphasizing practical, hands-on competencies. Roles requiring a “high school diploma or above” accounted for 9.58% of the postings; these positions were mostly in management or required professional qualifications, such as head nurse or care team leader. Such positions usually involve responsibilities in team management or providing professional nursing guidance.

**Table 2:** Basic thresholds for older adult care workers.

Variable	Category	Frequency	Percentage (%)
Work experience	No experience required	2,360	76.92
	1–3 years of experience	442	14.41
	< 1 year	174	5.67
	More than 3 years	92	3.00
Educational requirements	No minimum requirement	2,452	79.92
	Junior high or below	322	10.50
	High school or above	294	9.58
Total		3,068	100.00

Table 3 presents that the average monthly salary for older adult care workers was RMB 6,813.04. When categorized by regional economic status, the mean monthly salaries in high-, medium-, and low-level economic regions were RMB 7,368.66, RMB 6,305.01, and RMB 6,084.35, respectively. As the data violated the assumption of homogeneity of variance, Welch's ANOVA was conducted. ANOVA revealed that the difference in mean monthly salaries across the economic tiers was statistically significant (*P* < 0.001), demonstrating that salary levels vary significantly with regional economic development, with more developed regions offering higher remuneration.

**Table 3:** Differences in salary levels of older adult care workers by region and economic level.

Variable	Category	Sample size	Mean	Standard deviation	Welch's ANOVA
Monthly salary	High	1,589	7,368.66	2,231.35	*F* = 122.258 *P* = 0.000^***^
	Medium	883	6,305.01	1,939.06	
	Low	596	6,084.35	1,856.19	
Total	3,068	6,813.04	2,159.88		

^***^p < 0.001.

### 3.3 Core competency requirements

According to the frequency and proportion of each competency requirement in recruitment postings, the demand for older adult care job competencies, as presented in Table 4, can be summarized as follows.

**Table 4:** Skill categories mentioned in older adult care job advertisements.

Variable	Category	Frequency	Percentage (%)
Knowledge	Older adult care knowledge	969	31.58
	Certification	629	20.50
Daily living support skills	Personal hygiene assistance	2,155	70.24
	Domestic maintenance	1,924	62.71
	Mealtime assistance	1,838	59.91
	Mobility and transfer assistance	1,449	47.23
	Safety precautions	666	21.71
Health care skills	Routine health monitoring and care	1,284	41.85
	Rehabilitative care	657	21.42
	Emergency response	531	17.31
Psychosocial support skills	Emotional companionship	804	26.21
	Social-recreational support	301	9.81
General ability	Communication skills	1,265	41.23
	Service awareness	430	14.02
Traits	Patience	979	31.91
	Compassion	893	29.11
	Conscientiousness	877	28.59
	Attentiveness	325	10.59
Motives and values	Dedication	420	13.69
	Passion for older adult care	367	11.96
	Respect and dignity	197	6.42
Other requirements	In good physical health	860	28.03
	Proficient in smartphone use	223	7.27
	Can ride an electric bike	111	3.62

Daily living support skills were the most frequently required by employers, with personal hygiene assistance the highest at 70.24%, followed by domestic maintenance (62.71%) and mealtime assistance (59.91%). Mobility and transfer assistance (47.23%) and safety precautions (21.71%) were also frequently required. Regarding health care skills, routine health monitoring and care were in greatest demand (41.85%), followed by rehabilitative care (21.42%) and emergency response (17.31%). Psychosocial support focused mainly on emotional companionship (26.21%), whereas social-recreational support was less in demand (9.81%).

For general abilities, communication skills were prominently required at 41.23%, much higher than service awareness (14.02%). Regarding personal traits, patience (31.91%), compassion (29.11%), and conscientiousness (28.59%) were the top three requirements, whereas attentiveness accounted for 10.59%. Regarding motives and values, dedication (13.69%) and passion for older adult care (11.96%) were more frequently emphasized than respect and dignity (6.42%).

Regarding knowledge and certification, 31.58% of postings required older adult care knowledge, and 20.50% specified formal certification (older adult care or nursing licenses). Among other requirements, good physical health was essential (28.03%), whereas proficiency in smartphone use (7.27%) and the ability to ride an electric bike (3.62%) were the least frequently requested.

### 3.4 Factors associated with high salary

The binary logistic regression analysis (Table 5) revealed that the positive predictors of high salaries for older adult care workers included urban economic level (*B* = 0.770, OR = 2.160, 95% CI: 1.890–2.468, *P* < 0.001), compassion (*B* = 0.409, OR = 1.505, 95% CI: 1.230–1.842, *P* < 0.001), communication skills (*B* = 0.389, OR = 1.476, 95% CI: 1.215–1.791, *P* < 0.001), and mealtime assistance skills (*B* = 0.316, OR = 1.372, 95% CI: 1.099–1.712, *P* = 0.005). These findings indicate that higher urban economic levels and the possession of the aforementioned skills and traits by care workers are associated with a significantly increased probability of attaining high salaries.

**Table 5:** Binary logistic regression analysis of factors associated with high salaries.

Variables	*B*	S.E.	Wald χ^2^	*p*	OR (95% CI)
Work experience	−1.479	0.169	76.297	< 0.001	0.228 (0.164–0.317)
Educational requirement	−0.754	0.155	23.682	< 0.001	0.470 (0.347–0.638)
Older adult care knowledge	−0.238	0.109	4.78	0.029	0.788 (0.637–0.976)
Certification	−0.417	0.134	9.729	0.002	0.659 (0.507–0.857)
Personal hygiene assistance	−0.426	0.121	12.404	< 0.001	0.653 (0.515–0.828)
Mealtime assistance	0.316	0.113	7.796	0.005	1.372 (1.099–1.712)
Communication skills	0.389	0.099	15.574	< 0.001	1.476 (1.215–1.791)
Compassion	0.409	0.103	15.804	< 0.001	1.505 (1.230–1.842)
Conscientiousness	−0.531	0.114	21.761	< 0.001	0.588 (0.470–0.735)
Urban economic level	0.77	0.068	129.327	< 0.001	2.160 (1.890–2.468)
Constant	−1.889	0.144	173.03	< 0.001	—

Conversely, the negative predictors comprised work experience (*B* = −1.479, OR = 0.228, 95% CI: 0.164–0.317, *P* < 0.001), educational requirements (*B* = −0.754, OR = 0.470, 95% CI: 0.347–0.638, *P* < 0.001), and conscientiousness (*B* = −0.531, OR = 0.588, 95% CI: 0.470–0.735, *P* < 0.001). This suggests that more stringent requirements for rigid thresholds, such as work experience and educational attainment in job positions, are inversely associated with the likelihood of high salaries, indicating that these positions place greater emphasis on humanistic literacy and practical operational skills rather than on traditional qualification-based thresholds.

## 4 Discussion

This study analyzed the expectations of employers regarding the core competencies of older adult care workers in older adult care institutions in China using automated content analysis and the iceberg model as the framework. Compared with traditional research relying on small sample questionnaires or interviews (26–28), this research is based on large-scale real recruitment data and can better capture the dynamic changes in actual market demand. The results show that the market demand for older adult care workers includes a multi-level competency framework. This not only reflects the ongoing demand for basic nursing services in the industry, but also highlights the increasingly significant trend toward the specialization and integration of this profession aimed at an aging population and the promotion of the “integrated medical and older adult care” policy.

First, the results confirmed that daily care skills are still the core foundation of the older adult care industry. The frequent provision of skills such as personal hygiene assistance, domestic maintenance, and mealtime assistance demonstrates that meeting the basic living needs of older adults remains the basic function of the industry. Similar to the findings of this study, McKittrick et al. conducted a latent class analysis on a routine older adult care assessment database at the population level and discovered that the needs of older adults for daily activities in the home and community were very common (29). In particular, the demand for assistance with household chores (over 93%), shopping (over 87%), and meal preparation (over 90%) is prominent. This aligns with the core principle of international LTC philosophy, which emphasizes ensuring the quality of daily life for older individuals. Furthermore, this study found that the demand frequency of employers for knowledge among older adult care workers was lower than for the living care skills dimension. This reflects the employment logic of “learning-by-doing” in China: Employers are more concerned with whether the care tasks can be completed immediately than with a systematic reserve of gerontology knowledge. Employer requirements may differ by institution type, with LTC institutions focusing more on skills (30), whereas specialized care (dementia care) may emphasize professional knowledge (31).

Second, the expectations of employers for the capabilities of older adult care workers have significantly exceeded those of traditional “nanny-style” care. This study found that the demands of the employers for Health Care Skills, such as Routine Health Monitoring and Care (41.85%), Rehabilitative Care (21.42%), and Emergency Response (17.31%), far exceed the role boundaries of traditional daily living care workers. This is in line with the “integrated medical and care competencies” demands discovered by Wendt et al. (32). This indicates that Chinese employers are assigning specific primary health management responsibilities to the older adult care workers to compensate for shortages in the primary healthcare workforce. Consequently, the role of older adult care workers is transitioning from a daily living assistant to a “frontline health gatekeeper” equipped with basic health management capabilities. In an aging society, it is evident that society needs to cultivate dedicated medical personnel to serve the field of older adult care to meet community needs (33). This finding aligns with the issue of a “mismatch between continuing education provision and the genuine requirements of older adult care workers” identified by Martyn et al. (10), thereby suggesting that higher levels of older adult care workers should further improve modules of the latter, including rehabilitation assessment and chronic disease examination, to cope with the structural mismatch of labor supply and demand. Some organizations have initiated “certification subsidies” or paid training, exemplifying a proactive response to this emerging trend. The increasing significance of psychosocial support skills is evident. The recurrent appearance of keywords such as emotional companionship (26.21%) indicates a transition in quality care from a task-oriented to a relationship-oriented model. Employers are increasingly recognizing the importance of the ability of older adult care workers to build trusting relationships with older adults and provide emotional support.

Furthermore, soft skills and humanistic care literacy have become key elements in evaluating the comprehensive competence of care workers, and are also important factors in earning a high salary. The positive predictive effect of humanistic qualities such as compassion and communication skills on high salaries highlights the core trend of the older adult care industry shifting from basic to humanistic care, which aligns strongly with the philosophy of person-centered care (34, 35). Compassion is regarded as the foundation for delivering humanized, dignified care. Multiple studies have shown that compassion not only influences the quality of care but is also closely associated with the physical and mental wellbeing of both caregivers and care recipients (36–38). Employers are willing to pay a premium for such “hidden value,” which essentially reflects recognition of the fundamental nature of care as relationship-building. Meanwhile, older adult care is a job that relies heavily on interpersonal interactions. Effective communication is essential for understanding individual needs and improving service quality (39, 40). Multiple studies on competency assessment tools for older adult care have listed communication skills as core dimensions (41). However, in practice, many frontline older adult care workers lack sufficient communication or cross-cultural communication skills. Another key finding of this study is that conventional rigid employment thresholds, including work experience and educational background, have a significant negative predictive effect on high wages for older adult care workers. This diverges from the traditional labor market belief that seniority is positively associated with remuneration (42, 43). This can be attributed to the fact that high-paying positions in the current older adult care industry place greater emphasis on job suitability and practical service capabilities than formalized seniority requirements.

While digital transformation advances in older adult care (44), job postings rarely specify digital competence, and only a limited number of ads explicitly require smartphone proficiency from candidates. This indicates that digital literacy is rarely explicitly required for recruitment to older adult care positions. The digital literacy of older adult care workers is generally low (45). Smartphones are key tools for improving digital literacy (46), and expanding operational proficiency in their use will be crucial to optimizing service effectiveness.

It needs to be clarified that job advertisements reflect employer-stated preferences shaped by multiple contextual factors (platform templates, organizational communication styles, recruitment strategies, and local labor market conditions), rather than the full set of core competencies. All claims are scoped to employer-defined requirements to avoid overextension. Future multi-source validation is needed to confirm actual core competencies.

## 5 Conclusions

Based on a large-scale recruitment of the older adult care workers, this study presents empirical evidence that employer-defined competency demands for older adult care workers are evolving, shifting from a focus on daily living services to a care-centered, integrated model including daily care services, health management, and psychosocial support. Soft skills and humanistic care literacy are key to evaluating the comprehensive competence of care workers and vital for securing high salaries. The shift speaks to an aging population with rapidly increasing demand for complex care, as well as the general direction toward professionalization and occupationalization of the sector.

This study has some limitations. First, the urban sampling scheme stratified regions exclusively by per capita GDP, failing to fully incorporate other critical determinants of demand for older adult care labor, such as population aging structure, city size, and the maturity of local older adult care industries. This compromises the representativeness and generalizability of the sample. Second, the data were mainly retrieved from mainstream online recruitment platforms. As such, they may fail to capture the real needs of small and medium-sized institutions in underserved areas that primarily use offline recruitment channels, potentially leading to selection bias. Furthermore, the data collection only spanned two consecutive months. Subject to short-term recruitment volatility and seasonal variations, the dataset cannot fully reflect the long-term and stable requirements for the competencies of older adult care workers.

Future research could expand data sources and include longitudinal tracking to more comprehensively understand labor market dynamics and eliminate the influence of short-term fluctuations, so as to provide more robust empirical evidence for the industry. This study primarily adopted the perspective of the employer; however, subsequent investigations should integrate the perspectives of care workers and care recipients to provide a more diverse and comprehensive framework for older adult care.

## Data Availability

The raw data supporting the conclusions of this article will be made available by the authors, without undue reservation.
