# Supplementary material for: Understanding core competencies of older adult care workers from the perspectives of the employer: automated content analysis of job advertisements
Source: Front Public Health. 2026 Aug 18;14:1847475. doi: 10.3389/fpubh.2026.1847475 (PMC13529555; doi:10.3389/fpubh.2026.1847475)
Supplement: Supplementary file 1 [file Supplementary_file_1.docx]

[] 1.知识

<prompt=指与养老照护相关的理论和实践信息，包括对养老照护的基本理解、原则和方法。>

养老照护相关知识

<query=("知识") or ("相关专业") or ("经验者优先") or ("以上工作经验") or ("以上护理经验") or ("护理专业") or ("了解常见") or ("工作经验优先") or ("有一定的工作经验") or ("从业经验") or ("背景") or ("陪护经验") or ("相关法律法规") or ("相关工作经验") or ("经验优先") or ("有护理经验")>

<prompt=涉及关于老年人照护的生活、健康、心理及社会支持等方面的知识，帮助照护者提升对老年人需求的认识。>

资格证书

<query=("证书") or ("护理证") or ("资格证") or ("资质证书") or ("护工证") or ("岗位证") or ("上岗证") or ("护理员资质") or ("护理员证")>

<prompt=指在养老照护领域中，经过培训和考核获得的正式证明，表明持证者具备专业的知识和技能，可以从事养老照护工作。>

[] 2.技能-日常生活照料

个人卫生护理

<query=("身体清洁") or ("洗漱") or ("生活照料") or ("助浴") or ("如厕") or ("排泄") or ("个人卫生") or ("仪表仪容") or ("日常") or ("照料") or ("起居")or ("日常生活")or ("洗澡")or("洗脸") or ("洗脚")or ("剪指甲")or ("穿")or ("清洁")or ("衣食住行")or ("生活起居")>

<prompt=包括沐浴、洗脸、洗手、口腔护理、刷牙、头发护理、指甲修剪等。>

环境清洁

<prompt=包括为老年人搭配衣物、选择适合老年人的服饰，以及给予老年人适当的化妆和头发造型等护理。>

<query=("打扫") or ("环境") or ("房间") or ("更换床单") or ("搞卫生") or ("起居") or ("区域") or ("消毒")or ("日常生活")or ("衣食住行")or ("生活照料")or ("生活起居")>

饮食照护

<prompt=包括为老年人制定合理的饮食计划，合理搭配食物，确保老年人的营养需求得到满足。>

<query=("日常膳食") or ("饮食") or ("吃饭") or ("喂饭") or ("进食") or ("餐饮") or ("饭") or ("鼻饲") or ("做饭")or ("日常生活")or ("衣食住行")or ("生活照料")or ("生活起居")or ("膳食")>

安全防护

<query=("安全") or ("防护") or ("跌倒") or ("走失") or ("防范") or ("风险") or ("意外事故") or ("意外")>

日常活动协助

<query=("遛弯") or ("散步") or ("锻炼") or ("翻身") or ("拍背") or ("协助移动") or ("室内外活动") or ("运动") or ("活动") or ("外出")or ("助行")or ("衣食住行")or ("生活照料")or ("生活起居")>

[] 3.技能-健康护理技能

日常健康监测与护理

<prompt=包括为老年人管理慢性病症状，如高血压、糖尿病等，包括定时给药、监测血压和血糖、协助老年人进行相关的常规检查等。>

<query=("血压") or ("血糖") or ("慢性病") or ("体温") or ("记录") or ("定期测量") or ("脉搏") or ("呼吸") or ("评估") or ("观察") or ("采集")or ("监测")or("健康护理") or ("医疗护理") or ("喂药") or ("医嘱") or ("基础护理") or ("压疮") or ("用药") or ("护理操作")or ("消毒")or ("基本护理")or ("配合医生")or ("疾病护理") >

康复护理

<prompt=包括为老年人进行日常的体位调整、按摩、康复练习等，帮助老年人预防和改善身体功能障碍。>

<query=("康复") or ("功能训练") or ("药物")or ("训练")or ("理疗")or ("按摩")or ("物理治疗")or ("针灸")>

突发情况处理

<prompt=包括心肺复苏、出血处理、骨折处理等急救技能，以保障老年人在突发状况下的安全。>

<query=("紧急") or ("急救") or ("突发情况") or ("异常")or ("救急")or ("救援")or ("突发")>

[] 4.技能-心理关怀

精神慰藉

<prompt=包括在老年人情绪低落、孤独等情况下给予适当的安慰和陪伴，帮助老年人建立积极的心态。>

<query=("安慰") or ("陪伴") or ("心理照护") or ("情绪疏导") or ("精神照顾") or ("情感支持") or ("情绪") or ("关怀") or ("陪老人") or ("缓解焦虑") or ("精神慰藉") or ("关爱") or ("心理") or ("心情") or ("心理慰藉") or ("临终关怀")>

协助老年人文娱活动

<prompt=包括向老年人提供有效的解决问题的方法和建议，帮助老年人改变不良情绪和行为。>

<query=("文娱活动") or ("娱乐") or ("社交") or ("唱歌") or ("跳舞") or ("手工") or ("精神文化生活") or ("集体活动") or ("打牌") or ("游戏") or ("书法") or ("绘画") or ("节日活动")>

[] 5.社会角色与价值观

尊重老人

<query=("尊重") or ("尊敬") or ("尊严") or ("敬老") or ("尊老")>

服务意识

<query=("服务意识") or ("态度好") or ("态度端正")>

[] 6.个性与特质

<prompt=指在职业活动中，个体所需具备的专业知识、技能与态度的综合体现。>

责任心

<prompt=指个体在工作中对自己职责的自觉承担和履行，表现出对任务完成质量和后果的关注与重视。>

<query=("责任心") or ("责任感") or ("负责任") or ("尽心负责")>

爱心

<prompt=指个体在职业活动中表现出的对他人关怀与同情的情感，能够理解他人的需求并愿意提供帮助。>

<query=("爱心") or ("关爱")>

耐心

<prompt=指个体在面对挑战、困难或他人行为时，保持冷静和坚持，不轻易放弃，能够细致、周到地处理各类问题。>

<query=("耐心")>

细心

<query=("细心") or ("细致")>

<prompt=指个体在工作中对细节的关注和处理能力，及时发现问题并进行纠正，确保工作质量。>

抗压能力

<query=("抗压") or ("压力")>

沟通交流能力

<query=("交流") or ("沟通") or ("聊天") or ("陪聊")>

[] 7.动机与内驱力

奉献精神

<query=("奉献") or ("吃苦耐劳") or ("吃苦")>

热爱养老事业

<query=("热爱") or ("爱岗") or ("敬业") or ("认同") or ("有志于从事") or ("投身养老") or ("事业心") or ("有意愿从事养老事业") or ("有意愿从事养老行业")>

[] 8.其他要求

<prompt=指求职者在工作中需具备的特定条件或技能。>

会使用智能手机

<query=("智能手机") or ("手机") or ("微信")or ("导航")or ("地图")or ("照片")or ("智能")>

<prompt=指求职者具备使用智能手机进行基本操作的能力，包括拨打电话、发送信息、使用应用程序等。>

有健康证明

<query=("身体健康") or ("健康证明") or ("健康证")>

<prompt=指求职者提供的由专业医疗机构出具的健康状况证明，表明其符合健康要求。>

会骑电瓶车

<query=("电动车") or ("电瓶车") or ("骑车")>

<prompt=指求职者能熟练掌握电瓶车的骑行技巧，具备安全骑行的能力。>
